# Susceptibility of livestock-associated methicillin-resistant *Staphylococcus aureus* (LA-MRSA) to chlorhexidine digluconate, octenidine dihydrochloride, polyhexanide, PVP-iodine and triclosan in comparison to hospital-acquired MRSA (HA-MRSA) and community-aquired MRSA (CA-MRSA): a standardized comparison

**DOI:** 10.1186/s13756-019-0580-9

**Published:** 2019-07-22

**Authors:** Kathleen Dittmann, Thomas Schmidt, Gerald Müller, Christiane Cuny, Silva Holtfreter, Daniel Troitzsch, Peter Pfaff, Nils-Olaf Hübner

**Affiliations:** 1 Institute of Hygiene and Environmental Medicine, University Medicine of Greifswald, Walther-Rathenau-Str. 49a, 17489 Greifswald, Germany; 2 Robert Koch Institute, Unit 13: Nosocomial Pathogens and Antibiotic Resistances, Burgstraße 37, 38855 Wernigerode, Germany; 3 Department of Immunology, University of Greifswald, Ferdinand-Sauerbruch-Str, 17475 Greifswald, Germany; 4 BBraun AG, Carl-Braun-Straße 1, 34212 Melsungen, Germany; 5 University Medicine of Greifswald, Institute of Hygiene and Environmental Medicine, Ferdinand-Sauerbruch-Straße, 17475 Greifswald, Germany

**Keywords:** MRSA, Resistance, Decolonization, Antisepsis

## Abstract

**Background:**

Recent publications have raised concerns of reduced susceptibilities of clinical bacterial isolates towards biocides. This study presents a comparative investigation of the susceptibility of livestock-associated Methicillin-resistant *Staphylococcus aureus* (LA-MRSA), hospital-acquired MRSA (HA-MRSA) and community-aquired MRSA (CA-MRSA) to the commonly used antiseptics chlorhexidine (CHX), octenidine (OCT), polyhexanide (PHMB), PVP-iodine (PVP-I) and triclosan (TCX) based on internationally accepted standards.

**Methods:**

In total, 28 (18 LA-, 5 HA- and 5 CA) genetically characterized MRSA strains representing a broad spectrum of hosts, clonal complexes and spa-types, as well as the reference methicillin-sensitive *Staphylococcus aureus* (MSSA) strain ATCC 6538, were selected. Minimal inhibitory concentration (MIC) and minimal microbicidal concentration (MBC) were determined in accordance with DIN 58940–7, 58940–8 and DIN EN ISO 20776-1. The microbicidal efficacy was determined in accordance with DIN EN 1040.

**Results:**

Results from the MIC/MBC and quantitative suspension tests revealed differences between antiseptic substances but not between epidemiological groups of MRSA strains. OCT and PHMB were the most active substances with a minimal MIC of 1 mg/L, followed by CHX (2 mg/L), TCX (32 mg/L) and finally PVP-I (1024 mg/L). The MSSA reference strain showed a tendency to a higher susceptibility compared to the MRSA strains.

**Conclusions:**

This investigation of the susceptibility of a range of LA-, HA- and CA-MRSA strains using standardized conditions gave no indication that LA-MRSA strains are less susceptible to commonly used antiseptics compared to HA- and CA-MRSA strains.

## Background

Antiseptic agents such as chlorhexidine digluconate (chlorhexidine, CHX), octenidine dihydrochloride (OCT), polyhexanide (polyhexamethylene biguanide, PHMB), PVP-iodine (Poly(vinylpyrrolidone)-iodine complex, PVP-I), and triclosan (5-chlorine-2-(2,4-dichlorphenoxy)-phenol, TCX) are widely used as topical antiseptics against colonization and infection of humans and animals with Methicillin-resistant *Staphylococcus aureus* (MRSA) [1–7]. The clinical benefits of decolonization of MRSA patients for prevention of nosocomial infections is well documented [8–10]. The antimicrobial properties of these agents against hospital acquired (HA) MRSA strains have been repeatedly shown [11–17]. However, to our best knowledge, there are no systematic investigations comparing the susceptibility of livestock-associated (LA) and community-associated (CA) strains versus HA-MRSA strains to these antiseptics using standardized and harmonized test procedures. As CA- and LA-MRSA strains make up a growing proportion of MRSA strains in humans [18], such studies are quite pertinent. Our investigation was to test different antiseptics to selected MRSA strains reflecting stains that are prevalent in Germany with the main attention on LA-MRSA.

## Methods

In order to provide reliable and reproducible information on the susceptibility of MRSA strains, the minimal inhibitory concentration (MIC), the minimal microbicidal concentration (MBC) (microdilution test; EN 58940) as well as the microbicidal efficacy (quantitative suspension test; EN 1040) of CHX, OCT, PHMB, PVP-I and TCX were determined in a comparative study under standardized conditions [19–22] using a spectrum of genetically characterized strains from different hosts.

### Test strains

Strains were drawn from the national collection of the Robert Koch-Institute (RKI) to represent a broad spectrum of strains from different hosts that are prevalent in Germany, and from a collection of regional strains from northeastern Germany (HICARE Study) (Table 1) [23]. Methicillin-sensitive *Staphylococcus aureus* (MSSA) ATCC 6538 was used as the reference strain. The reference strain comes from the American Type Culture Collection (ATCC®), a scientifically recognized source, and has defined resistance properties [24].

**Table 1:** List of LA-MRSA, HA-MRSA and CA-MRSA strains with source, spa-type, SCCmec, resistance phenotype and provider

LA-MRSA	Source	*Spa-*type	SCCmec; other	Resistance Phenotype	Provider
CC398	pig	t034	V	PEN, OXA, ERY, CLI, TET, CIPi, SXT, OXA/Su	RKI
CC398	cow	t011	ND	PEN, OXA, TET, OXA/Su	RKI
CC398	turkey	t034	ND	PEN, OXA, ERY, CLI, TET, SXTi, OXA/Su	RKI
CC398	poultry	t011	ND	PEN, OXA, ERY, CLI, TET, SXT, OXA/Su	RKI
CC398	horse	t011	IV	PEN, OXA, GEN, ERY, CLI, TET, CMP, SXT, OXA/Su	RKI
CC398	horse	t6867	IV	PEN, OXA, GEN, TET, COX, OXA/Su	RKI
CC398	human	t034	V	PEN, OXA, TET, SXTi, OXA/Su	RKI
CC398	human	t899	IV	PEN, OXA, OXA/Su	RKI
CC398	human	t2123	ND	PEN, OXA, GEN, TET, CIP, OXA/Su	RKI
CC398	human	t2370	ND	ND	HICARE
CC398	human	t1456	ND	ND	HICARE
CC398	human	t3275	ND	ND	HICARE
CC398	human	t10721	ND	ND	HICARE
CC130	deer	t843	ND	PEN, OXA, OXA/Su	RKI
CC130	horse	t843	ND	PEN, OXA, OXA/Su	RKI
CC130	human	t1773	ND	PEN, OXA, CIPi, OXA/Su	RKI
CC9	chicken	t1430	ND	PEN, OXA, CIP, MFL, OXA/Su	RKI
CC9	human	t1430	ND	PEN, OXA, ERY, CLI, CIP, MFL, OXA/SU	RKI
HA-MRSA	Source	*Spa*-type	SCCmec; other		Provider
CC22	human	t032	IV	PEN, OXA, ERY, CLI, CMP, CIP, MFL, OXA/Su	RKI
CC22	human	t020	ND	PEN, OXA, ERY, CLI, CIP, MFL, OXA/Su	RKI
CC22	human	t005	ND	PEN, OXA, OXA/Su	RKI
CC5 (ST225)	human	t003	II	PEN, OXA, ERY, CLI, CMP, CIP, MFL, OXA/Su	RKI
CC5 (ST5)	human	t002	ND	PEN, OXA, ERY, CLI, CIP, MFL, OXA/Su	RKI
CA-MRSA	Source	*Spa*-type	SCCmec; other		Provider
CC1	human	t5100	nd, lukPV, seh	PEN, OXA, GEN, TET, FUS, COX, OXA/Su	RKI
CC8	human	t1476	ND	ND	HICARE
CC8	human	t008	IV, lukPV	PEN, OXA, ERY, CIP, MFL, OXA/Su	RKI
CC80	human	t044	IV, lukPV	PEN, OXA, TET, CIP, MUPi, FUS, OXA/Su	RKI
CC59	human	t437	nd, lukPV	PEN, OXA, ERY, CLI, TET, CMP, OXA/Su	RKI

*RKI* Robert Koch-Institute, *HICARE* HICARE Study, *ND* not determinded; Groups of strains were defined genetically by spa-typing, MLST, and SCC*mec,* as well as demonstration of *luk*-PV

The *S. aureus* Genotyping Kit 2.0 (Alere Technologies GmbH, Jena, Germany) was used to test selected isolates for the presence of genes encoding quaternary ammonium compound efflux pumps (*qac* genes), as described elsewhere [25]. None of the tested strains in this comparison harbored *qac* genes.

### Test preparations

Chlorhexidine digluconate (20% CHX solution, C 9394, Sigma-Aldrich Biochemie GmbH, Hamburg, Germany), octenidine dihydrochloride (Schülke & Mayr GmbH, Norderstedt, Germany), polyhexanide (20% PHMB solution, Fagron GmbH & Co. KG, Hamburg, Germany) and PVP-I (Betaisodona solution: 100 ml of the solution contains 10 g of poly(1-vinyl-2-pyrrolidone-)iodine-complex, with a content of 11% available iodine, Mundipharma GmbH, Limburg, Germany) and were diluted in water of standardized hardness (WSH; according to DIN EN 1040 [19]) to the final test concentrations. As TCX (Irgasan, 72779, Fluka, Buchs, Switzerland) dissolves poorly in water, a stock solution of 50% TCX in 80% dimethylsulfoxide (DMSO) was diluted in several steps to obtain a final concentration of 1% TCX in 40% DMSO/WSH. A 40% DMSO/WSH solution was used in all dilution steps.

The suitability of 40% DMSO in WSH was demonstrated using the quantitative suspension test and the microdilution test as described previously [26].

The following solutions were used as neutralizing agents in accordance with DIN EN 1040 and 1275 [19, 20]:

- 3.0% (w/v) polysorbate 80 + 3.0% (w/v) saponin + 0.1% (w/v) L-histidine + 0.1% (w/v) cysteine for neutralizing CHX, OCT and PHMB
- 3.0% (w/v) polysorbate 80 + 0.3% (w/v) lecithin + 0.3% (w/v) L-histidine + 0.5% (w/v) sodium thiosulfate for neutralizing PVP-I
- 8.0% (w/v) polysorbate 80 + 2.0% (w/v) sodium dodecylsulfate (SDS) + 0.8% (w/v) lecithin + 1.0% (w/v) sodium thiosulfate + 6.0% (w/v) saponin for neutralizing TCX.

To determine the MICs and the MBCs, the substances were prepared in concentrations from 0.25 to 4096 mg/L (Table 2). Concentration ranges used in the quantitative suspension tests are summarized in Table 2.

**Table 2:** Concentration ranges used for determining MICs and MBCs in accordance with DIN EN 58940–7 and 58940–8) [18] and concentration ranges of the test preparations used in the quantitative suspension tests according to DIN EN 1040 [15]

CHX	OCT	PHMB	PVP-I	TCX
Concentration range of Antiseptic agent for MICs and MBCs determination [mg/L]				
0.25–4	0.25–4	0.25–4	256–4096	16–256
Concentration range of Antiseptic agents for quantitative suspension tests [mg/L]				
125–500	20–40	50–100	5,000 – 10,000	250–1,000

#### Microdilution test

DIN EN 58940–7 [21] and 58940–8 [22] and the corresponding supplementary sheets were strictly followed to determine the MIC and MBC, as described previously [26]*.* Briefly, the test organisms were cultivated on CASO agar at 37 °C for 18 h; thereafter, four to five colonies were transferred into 1 ml of BBL Mueller Hinton Broth (BD, Becton Dickinson) and diluted to reach 5 × 10^5^ cfu/ml. Tests were performed in 96-well microtiter plates. Each test was performed in duplicate. Each well was filled with 100 μl of defined antiseptic dilution and 100 μl of test organism suspension. The turbidity was visually evaluated as an indicator of bacterial growth and minimal inhibitory concentration after 24 h (MIC_24_) and after 48 h (MIC_48_). To determine the MBC, samples in the range of the threshold for turbidity after 24 h were transferred onto blood agar, as described in the standards, and evaluated for growth after 24 h incubation (MBK_24_).

### Quantitative suspension test

DIN EN 1040 [19] was strictly applied to determine the bactericidal efficacy without organic load. Briefly, 0.1 ml of test organism suspension and 0.1 ml of WSH were mixed and left for 2 min. Afterwards, 0.8 ml of the respective antiseptic test substance were added. The resulting solutions were incubated for 5 and 30 min at 37 °C. At the end of the contact time, 0.1 ml of the test solution was transferred to 0.8 ml of the respective neutralizing solution and 0.1 ml WSH and left for 5 min. Serial dilutions were prepared in neutralizer; 0.1 ml of each neutralized test dilution was spread onto nutrient agar plates in duplicates. After incubation for 24 h, the colonies were counted and the number of recoverable colonies (N_a_) in the test solution was calculated. The reduction factor (RF) was determined as the difference of the log number of cells in the test solution at the beginning of the contact time (N_0_) and log of N_a_.

In addition to the DIN EN, negative controls using 0.8 ml of WSH instead of test preparation were performed simultaneously in the first test run to exclude any bactericidal effects of WSH. In the water controls, no essential difference was observed compared to the N_0_ values.

### Statistics

Data were prepared using Microsoft Excel 2010 (Microsoft, Redmond, WA, USA) and analyzed using IBM SPSS Statistics 24 (IBM, Armonk, NY, USA). Strains were grouped as LA-, HA- and CA strains.

Robust nonparametric statistics were used to compare results from the microdilution tests and quantitative suspension tests [27]. A two-step procedure was chosen to avoid alpha-error inflation. Kruskal-Wallis tests were used as omnibus tests for multiple comparisons. If the omnibus tests indicated statistically significant differences between groups, Mann-Whitney tests were used for pairwise comparisons.

## Results

### MIC and MBC

Values of MICs and MBCs of tested substances showed marked differences between LA-, HA- and CA-MRSA (Table 3). OCT and PHMB were the most active substances with a minimum MIC of 1 mg/L followed by CHX, TCX and finally PVP-I. There was no significant difference between MIC_24_ and MIC_48_ of the same substances between LA-, HA- and CA-MRSA (Related-Samples-Wilcoxon-Signed-Rank Test, *p* = 1.00). There was a significant differences between MBC_24_ and MIC_24_ for all substances but TCX (Related-Samples-Wilcoxon-Signed-Rank-Test, *p* < 0.01). TCX showed the greatest range between minimum and maximum MIC and MBC values.

**Table 3:** Rounded means and range of MIC_24_, MIC_48_ and MBC LA-, HA- and CA-MRSA strains in mg/L

Stains	ATCC_6538 (reference)	LA-MRSA		HA-MRSA		CA-MRSA		Total		
Substance and Test	Mean	Mean	Range	Mean	Range	Mean	Range	Mean	Range	*p*-Value
CHX MIC _24_	4	2	2–4	3	2–4	3	2–4	3	2–4	0.217
CHX MIC_48_	4	2	2–4	3	2–4	3	2–4	3	2–4	0.217
CHX MBC_24_	8	5	4–8	4	4–4	5	4–8	5	4–8	0.173
OCT MIC _24_	2	1	1–2	1	1–1	1	1–2	1	1–2	0.153
OCT MIC_48_	2	1	1–2	1	1–1	1	1–2	1	1–2	0.153
OCT MBC_24_	4	2	2–4	2	2–2	3	2–4	3	2–4	0.153
PHMB MIC _24_	1	2	1–2	2	2–2	2	2–2	2	1–2	0.003
PHMB MIC_48_	1	2	1–2	2	2–2	2	2–2	2	1–2	0.003
PHMB MBC_24_	1	4	2–4	4	4–4	4	4–4	4	1–4	0.002
PVP-I MIC _24_	1024	1991	1024–4096	2048	2048–2048	2458	2048–4096	2048	1024–4096	0.098
PVP-I MIC_48_	1024	1991	1024–4096	2048	2048–2048	2458	2048–4096	2048	1024–4096	0.098
PVP-I MBC_24_	2048	3754	2048–4096	4096	4096–4096	4096	4096–4096	3814	2048–4096	0.053
TCX MIC _24_	8	52	32–64	64	64–64	102	64–256	61	8–256	0.020
TCX MIC_48_	8	52	32–64	64	64–64	102	64–256	61	8–256	0.020
TCX MBC_24_	8	52	32–64	64	64–64	102	64–256	61	8–256	0.020

Values of MIC_24_, MIC_48_ and MBC_24_ differed significantly between groups of strains for PHBM (*p* = 0.003, *p* = 0.003 and *p* = 0.002) and TCX (all *p* = 0.02) but not for CHX (*p* = 0.217, p = 0.217 and *p* = 0.173), OCT (all *p* = 0.153) and PVP-I (*p* = 0.098, p = 0.098 and *p* = 0.053) in the Independent-Samples Kruskal-Wallis test. Pairwise comparison of MIC_24_, MIC_48_ and MBC_24_ of PHMB showed that this was caused by a higher susceptibility of the reference MSSA strain compared to the MRSA strains (*p* < 0.02). Pairwise comparisons for TCX showed that HA- and CA-MRSA strains were less susceptible to TCX than the reference strain (*p* = 0.021 and 0.01 respectively), and CA-MRSA was significantly less susceptible than LA-MRSA (*p* = 0.035).

#### Quantitative suspension test

**Fig. 1:**
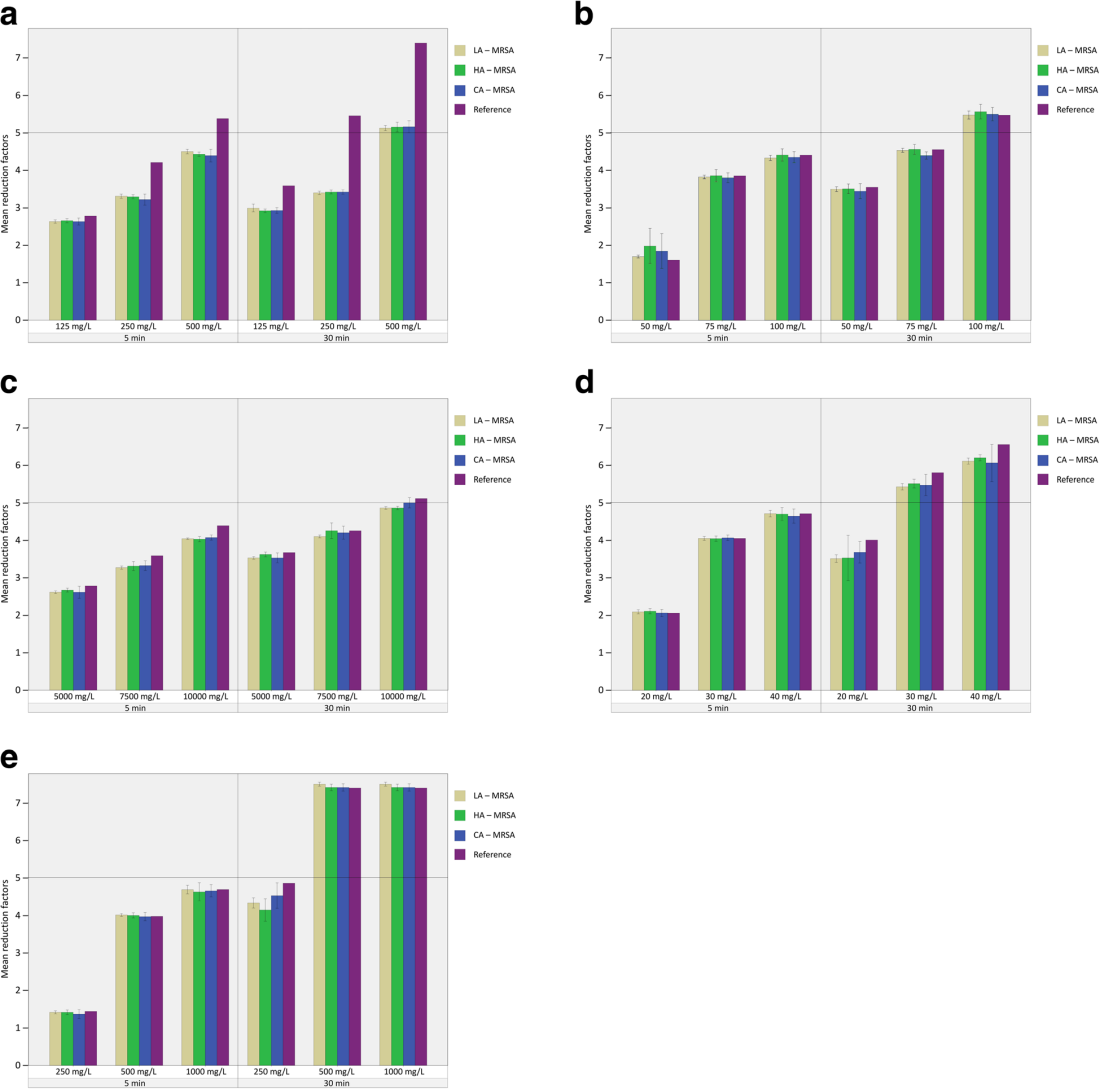
**a** Results of quantitative suspension test for chlorhexidine for LA-MRSA, HA-MRSA, CA-MRSA and reference MSSA. Different concentrations of chlorhexidine (CHX; 125 mg/L, 250 mg/L and 500 mg/L) were suspended to different MRSA strains and the MSSA reference strain at two different contact times (5 min and 30 min). LA-MRSA (beige), HA-MRSA (green), CA-MRSA (blue) and reference MSSA (purple). Error bars show 95% confidence intervals. Horizontal line at the value of the mean reduction factor of 5 indicates bactericidal threshold according to DIN EN 1040. **b** Results of quantitative suspension test for octinidine for LA-MRSA, HA-MRSA, CA-MRSA and reference MSSA. Different concentrations of octinidine (OCT; 50 mg/L, 75 mg/L and 100 mg/L) were suspended to different MRSA strains and the MSSA reference strain at two different contact times (5 min and 30 min). LA-MRSA (beige), HA-MRSA (green), CA-MRSA (blue) and reference MSSA (purple). Error bars show 95% confidence intervals. Horizontal line at the value of the mean reduction factor of 5 indicates bactericidal threshold according to DIN EN 1040. **c** Results of quantitative suspension test for polyhexanide for LA-MRSA, HA-MRSA, CA-MRSA and reference MSSA. Different concentrations of polyhexanide (PHMB; 5000 mg/L, 7500 mg/L and 10000 mg/L) were suspended to different MRSA strains and the MSSA reference strain at two different contact times (5 min and 30 min). LA-MRSA (beige), HA-MRSA (green), CA-MRSA (blue) and reference MSSA (purple). Error bars show 95% confidence intervals. Horizontal line at the value of the mean reduction factor of 5 indicates bactericidal threshold according to DIN EN 1040. **d** Results of quantitative suspension test for PVP-iodine for LA-MRSA, HA-MRSA, CA-MRSA and reference MSSA. Different concentrations of PVP-iodine (PVP-I; 20 mg/L, 30 mg/L and 40 mg/L) were suspended to different MRSA strains and the MSSA reference strain at two different contact times (5 min and 30 min). LA-MRSA (beige), HA-MRSA (green), CA-MRSA (blue) and reference MSSA (purple). Error bars show 95% confidence intervals. Horizontal line at the value of the mean reduction factor of 5 indicates bactericidal threshold according to DIN EN 1040. **e** Results of quantitative suspension test for triclosan for LA-MRSA, HA-MRSA, CA-MRSA and reference MSSA. Different concentrations of triclosan (TCX; 250 mg/L, 500 mg/L and 1000 mg/L) were suspended to different MRSA strains and the MSSA reference strain at two different contact times (5 min and 30 min). LA-MRSA (beige), HA-MRSA (green), CA-MRSA (blue) and reference MSSA (purple). Error bars show 95% confidence intervals. Horizontal line at the value of the mean reduction factor of 5 indicates bactericidal threshold according to DIN EN 1040

The archived reduction factors show that all substances were used at or below the concentration needed to achieve the threshold set by DIN EN 1040 (at least a 5 log-step reduction) to be adequately bactericidal, as planned. As expected, reduction factors increased with contact time and concentration of the antiseptic (Fig. 1a-e). In contrast, the MSSA reference strain showed a higher susceptibility to CHX than did the MRSA strains, but the differences were not statistically significant in the omnibus test. All other tests for statistical significance were omitted due to the small absolute differences and the overlapping confidence intervals.

## Discussion

The antibacterial activity of common antiseptics against a broad range of different pathogens has been well documented [26, 28]. Still, little is known about the differences in the susceptibility to antiseptics of LA-MRSA in comparison to HA-MRSA and CA-MRSA. While antiseptics show a broader antimicrobial spectrum compared to antibiotics and are less compromised by specific resistances, reduced susceptibility of various strains to antiseptics has been reported [29–33]. Besides antimicrobial agents other facts like metal-resistance genes might contribute to differences the susceptibility to antiseptics. For example Argudin et al. reported the occurrence of different metal-resistance genes among LA-MRSA [31]. Recent publications in particular have raised concerns of reduced susceptibilities of distinctive clinical isolates towards biocides and found associations with outbreaks [29, 33, 34].

Therefore, the susceptibility of LA-MRSA to antiseptics is an important issue, as LA-MRSA is an emerging problem and antiseptic agents are valuable drugs for prevention of MRSA infections [18, 35]. For example, antiseptic decolonization has been proven to control the spread of MRSA in intensive care healthcare settings [8] and to reduce surgical site infections [36]. Nevertheless, the effectiveness of these measures relies on the susceptibility of the targeted pathogens to the antiseptic products used.

Other mechanisms for reduced susceptibility to disinfectants in MRSA besides the qac gene coded efflux pumps have been described: reduced susceptibility to chlorhexidine can also result from mutations in the norA/norB genes which code for an efflux mechanism [29]. Reduced susceptibility to triclosan can be due to either enhanced expression of the target of this biocide, namely the enoyl-acyl carrier protein (ACP) reductase enzyme (FabI) [37], or acquisition of an additional sh-fabI allele derived from Staphylococcus haemolyticus by horizental gene transfer [27]. We found no evidence of reduced susceptibility of LA-MRSA to CHX, OCT, PHMB, PVP-I and TCX in comparison to CA- and HA-MRSA. Differences in the susceptibility between the strains in MIC, MBC and microdilution assays were marginal. With a difference not greater than one dilution step, the range between the highest and lowest MIC and MBC between the groups of MRSA stains was at the same level or even smaller, as between the strains of the same group (one step for CHX, PHMB and OCT and up to two steps for PVP-I and TCX). The only exception was TCX in terms of LA-MRSA strains, which were significantly more susceptible than CA-MRSA.

Likewise, the results from the quantitative suspension assays were quite comparable between CA-, HA- and CA-MRSA strains. In contrast, the reference MSSA strain showed a tendency to higher susceptibility in the MIC, MBC and quantitative suspension assays. However, as only one reference strain was used, it is unclear whether this can be interpreted as higher susceptibility of MSSA in contrast to MRSA or as an attribute of the specific strain.

Our results are well comparable with those of other published studies. MICs reported by Koburger et al. for aureus ATCC 6538 almost matched our results, with the exception of PVP-I and TCX, which showed a markedly higher MIC_48_ and MBC_24_ in our tests [26]. The differences for PVP-I remain unexplained, while the reported higher MICs to TCX in comparison to Koburger et al. (0.125 versus 8 mg/L) can be explained by the fact that 8 mg/L was the lowest concentration used in our tests.

Furthermore, the tested MRSA-strain, a northern German epidemic strain, showed susceptibilities comparable to our results. Likewise, MICs to PHMB and TXC reported by Assadian et al. for MRSA, low level vancomycin-resistant (VISA) *S. aureus* strains and *S. aureus* ATCC 29213 correspond well to our results [11]. Interestingly, the MSSA reference strains showed a tendency to higher susceptibility to TCX in this two studies compared to MRSA.

It is important to bear in mind that the concentrations used in our study were well below the concentrations recommended by the manufacturer. For example, PHMB is used at a concentration of 0.02% or 200 mg/L, which is 200 times greater than the MIC_24_ for wound antisepsis.

The strength of the present study is the systematic approach based both on European standards for assessing the bactericidal effects in quantitative suspension assays and on industry standards to determine the MIC and MBC using the microdilution method [19, 21, 22, 26]. Our method can therefore easily be replicated by other researchers and for other strains. One point worthy of note is that parts of DIN 58940 have since been suspended and replaced by DIN EN ISO 20776-1:2007–02. However, this has no effects in terms of determining the MIC and MBC for antiseptics in this study.

Our study has limitations. For instances, we used only a limited number of strains and antiseptics for our analysis. It is well known that some strains express higher resistances to specific antiseptics. Resistance to antiseptics can arise through different mechanisms [38]. For example, efflux-mediated resistance to various biocides linked to qac-genes has been reported in different staphylococcal isolates in recent years [39–41]. However, this does not detract from our research question of whether LA-strains show a higher resistance to antiseptics compared to HA- and CA strains per se, as qac-genes have been reported in HA-, CA- and LA-strains alike. Although we used a limited number of strains, all were genetically characterized and represented a broad spectrum of hosts, clonal complexes and spa-types. Most strains were drawn from the national collection of the Robert Koch Institute and were supplemented by regional strains from northeastern Germany as well as an ATCC reference strain. The aim of our study was to evaluate the susceptibility of LA-MRSA to different antiseptics in comparison to HA-MRSA and CA-MRSA. The MSSA reference strain serves as an intern control. The shown difference between the reference and the test strains should not be interpreted as evidence for a higher susceptibility of MSSA to MRSA strains in general.

Regarding the limited number of antiseptics used, we covered a broad spectrum of substances with different modes of action. Our selection included CHX, probably the most commonly used antiseptic agent worldwide, and OCT, PHMB, PVP-I and TCX. These substances are widely used in specific fields of application, such as antisepsis on skin and mucous membranes [1, 4], the eye [42, 43], acute and chronic wounds [2, 6] and sutures [44].

In summary, the present study gives no reason to doubt that the tested antiseptics can kill LA-MRSA at the concentrations recommended for use by the manufacturer. However, if the substances are diluted, which can happen deliberately as result of the usage (e.g., when irrigating wounds) or as part of the intended application (e.g., slow release of CHX from patches or TCX from sutures), the concentration may be reduced to levels that fall short of the MIC. As recent publications raise concerns about the increasing resistance of clinical isolates to antiseptics and disinfectants, this highlights the importance of safe and conscientious use of antiseptics.

## Conclusion

This investigation of the susceptibility of a broad range of HA-, LA- and CA-MRSA strains using standardized and harmonized conditions provided no indication that LA-MRSA strains show reduced susceptibility to commonly used antiseptics compared to HA- and CA-MRSA strains.

## Data Availability

The data and materials are available from the corresponding author on reasonable request.

## Abbrevations

CA-MRSA: Community-aquired Methicillin-resistant *Staphylococcus aureus*
CHX: Chlorhexidine
HA-MRSA: Hospital-acquired Methicillin-resistant *Staphylococcus aureus*
LA-MRSA: Livestock-associated Methicillin-resistant *Staphylococcus aureus*
MBC: Minimal microbicidal concentration
MIC: Minimal inhibitory concentration
MRSA: Methicillin-resistant *Staphylococcus aureus*
MSSA: Methicillin-sensitive *Staphylococcus aureus*
OCT: Octenidine
PHMB: Polyhexanide
PVP-I: (Poly(vinylpyrrolidone)-iodine complex)
TCX: Triclosan
