# Correction to: Susceptibility of livestock-associated methicillin-resistant Staphylococcus aureus (LA-MRSA) to chlorhexidine digluconate, octenidine dihydrochloride, polyhexanide, PVP-iodine and triclosan in comparison to hospital-acquired MRSA (HA-MRSA) and community-aquired MRSA (CA-MRSA): a standardized comparison

**DOI:** 10.1186/s13756-019-0601-8

**Published:** 2019-11-20

**Authors:** Kathleen Dittmann, Thomas Schmidt, Gerald Müller, Christiane Cuny, Silva Holtfreter, Daniel Troitzsch, Peter Pfaff, Nils-Olaf Hübner

**Affiliations:** 1 Institute of Hygiene and Environmental Medicine, University Medicine of Greifswald, Walther-Rathenau-Str. 49a, 17489 Greifswald, Germany; 2 RobertKoch Institute, Unit 13: Nosocomial Pathogens and Antibiotic Resistances, Burgstraße 37, 38855 Wernigerode, Germany; 3 Department of Immunology, University of Greifswald, Ferdinand-Sauerbruch-Str, 17475 Greifswald, Germany; 4 BBraun AG, Carl-Braun-Straße 1, 34212 Melsungen, Germany; 5 University Medicine of Greifswald, Institute of Hygiene and Environmental Medicine, Ferdinand-Sauerbruch-Straße, 17475 Greifswald, Germany

**Correction to: Antimicrob Resist Infect Control**

**https://doi.org/10.1186/s13756-019-0580-9**

The original article [1] contains an error in Fig. 1 whereby sub-figures 1B, 1C & 1D contain the wrong scale on the x-axis. The correct sub-figures are located ahead in this Correction article and should be considered instead.

**Fig. 1:**
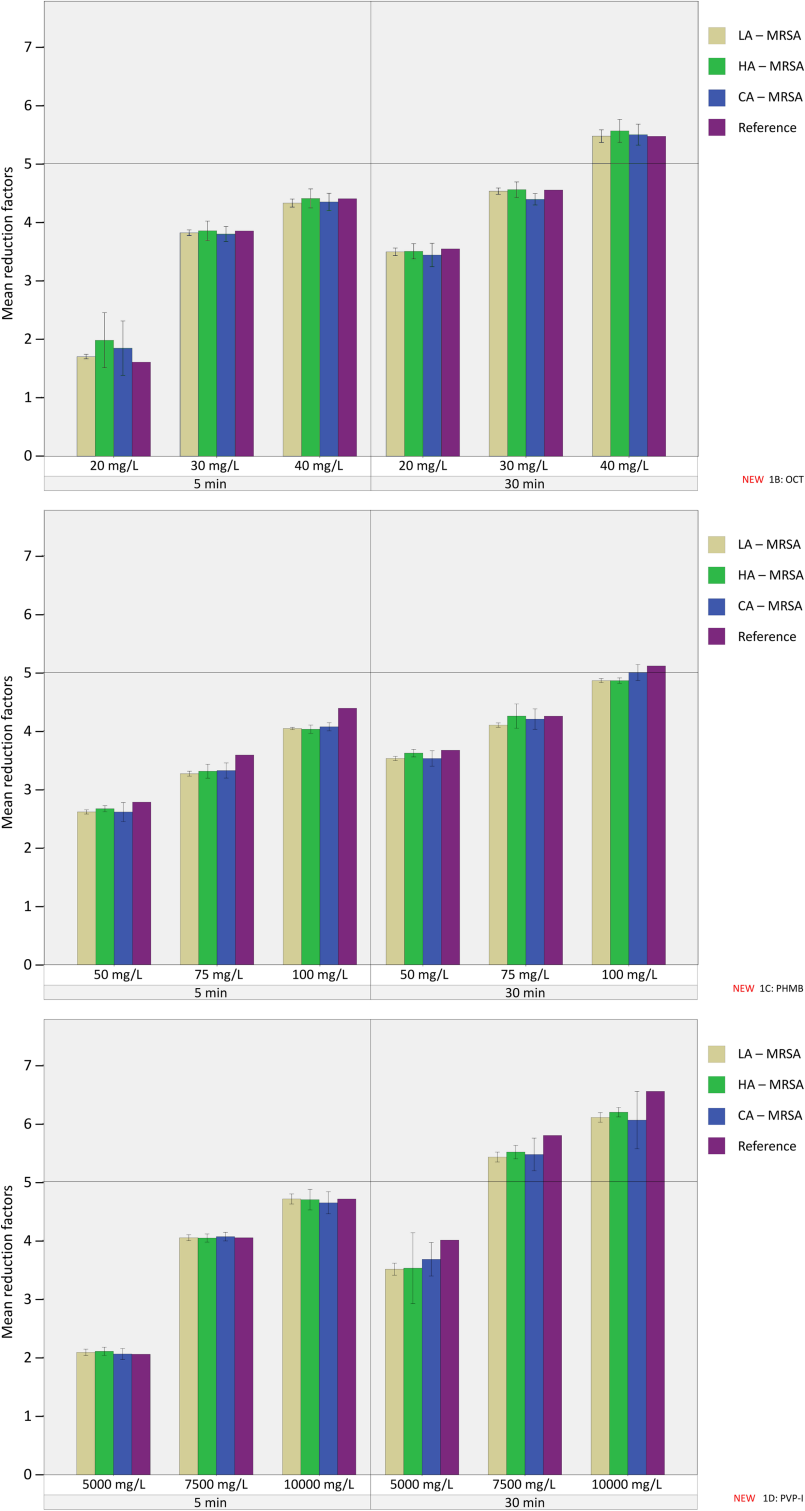
**a** Results of quantitative suspension test for chlorhexidine for LA-MRSA, HA-MRSA, CA-MRSA and reference MSSA. Different concentrations of chlorhexidine (CHX; 125 mg/L, 250 mg/L and 500 mg/L) were suspended to different MRSA strains and the MSSA reference strain at two different contact times (5 min and 30 min). LA-MRSA (beige), HA-MRSA (green), CA-MRSA (blue) and reference MSSA (purple). Error bars show 95% confidence intervals. Horizontal line at the value of the mean reduction factor of 5 indicates bactericidal threshold according to DIN EN 1040. **b** Results of quantitative suspension test for octinidine for LA-MRSA, HA-MRSA, CA-MRSA and reference MSSA. Different concentrations of octinidine (OCT; 50 mg/L, 75 mg/L and 100 mg/L) were suspended to different MRSA strains and the MSSA reference strain at two different contact times (5 min and 30 min). LA-MRSA (beige), HA-MRSA (green), CA-MRSA (blue) and reference MSSA (purple). Error bars show 95% confidence intervals. Horizontal line at the value of the mean reduction factor of 5 indicates bactericidal threshold according to DIN EN 1040. **c** Results of quantitative suspension test for polyhexanide for LA-MRSA, HA-MRSA, CA-MRSA and reference MSSA. Different concentrations of polyhexanide (PHMB; 5000 mg/L, 7500 mg/L and 10,000 mg/L) were suspended to different MRSA strains and the MSSA reference strain at two different contact times (5 min and 30 min). LA-MRSA (beige), HA-MRSA (green), CA-MRSA (blue) and reference MSSA (purple). Error bars show 95% confidence intervals. Horizontal line at the value of the mean reduction factor of 5 indicates bactericidal threshold according to DIN EN 1040. **d** Results of quantitative suspension test for PVP-iodine for LA-MRSA, HA-MRSA, CA-MRSA and reference MSSA. Different concentrations of PVP-iodine (PVP-I; 20 mg/L, 30 mg/L and 40 mg/L) were suspended to different MRSA strains and the MSSA reference strain at two different contact times (5 min and 30 min). LA-MRSA (beige), HA-MRSA (green), CA-MRSA (blue) and reference MSSA (purple). Error bars show 95% confidence intervals. Horizontal line at the value of the mean reduction factor of 5 indicates bactericidal threshold according to DIN EN 1040. **e** Results of quantitative suspension test for triclosan for LA-MRSA, HA-MRSA, CA-MRSA and reference MSSA. Different concentrations of triclosan (TCX; 250 mg/L, 500 mg/L and 1000 mg/L) were suspended to different MRSA strains and the MSSA reference strain at two different contact times (5 min and 30 min). LA-MRSA (beige), HA-MRSA (green), CA-MRSA (blue) and reference MSSA (purple). Error bars show 95% confidence intervals. Horizontal line at the value of the mean reduction factor of 5 indicates bactericidal threshold according to DIN EN 1040
